# Review on the Role of Polyphenols in Preventing and Treating Type 2 Diabetes: Evidence from In Vitro and In Vivo Studies

**DOI:** 10.3390/nu16183159

**Published:** 2024-09-19

**Authors:** Fereidoon Shahidi, Renan Danielski

**Affiliations:** Department of Biochemistry, Memorial University of Newfoundland, St. John’s, NL A1C 5S7, Canada; rdanielski@mun.ca

**Keywords:** enzymatic inhibition, cholesterol-lowering, gut microbiota, inflammation, oxidative stress

## Abstract

Type 2 diabetes (T2D) is one of the leading causes of death globally. There was a 70% increase in diabetes-related deaths between 2000 and 2020, particularly among males. This non-communicable disease is characterized by increased insulin resistance, leading to elevated blood sugar levels and, if untreated, resulting in complications such as nerve damage, kidney disease, blindness, and poor wound healing. T2D management includes dietary intervention, physical exercise, and the administration of blood sugar-lowering medication. However, these medications often have side effects related to intestinal discomfort. Therefore, natural alternatives to standard diabetes medications are being sought to improve the quality of life for individuals with this condition. Polyphenols, which are naturally occurring plant metabolites, have emerged as strong candidates for T2D control. Various phenolic acids (e.g., chlorogenic acid), flavonoids (e.g., quercetin), proanthocyanidins (e.g., procyanidin B2), gallotannins (e.g., monogalloyl hexoside), and ellagitannins (e.g., ellagic acid hexoside) can enhance insulin sensitivity in tissues, reduce chronic inflammation, scavenge free radicals, improve insulin secretion, inhibit enzymes involved in carbohydrate digestion, regulate glucose transport across cell membranes, and modulate gut microbiota. This contribution compiles up-to-date evidence from in vitro and in vivo studies on the role of polyphenols in the prevention and management of T2D, emphasizing the mechanisms of action underlying these effects.

## 1. Introduction

Type 2 diabetes (T2D) places ninth among the leading causes of death worldwide. It is estimated that approximately 537 million adults live with diabetes globally (predominantly T2D), with a projected increase of 46% by 2045. A demographic breakdown shows that from this total, approximately 80% of people with T2D are located in low-income and middle-income countries, exposing the economical inequalities related to the prevalence of this condition [1]. The onset of T2D is a reflection of lifestyle choices, where a combination of poor diet, lack of physical exercise, and damaged mental health contribute to a progressive increase in insulin resistance that can ultimately lead to T2D. Usually, this form of diabetes is associated with weight gain and obesity. In most cases, financial means dictate lifestyle choices, with accessibility and affordability of whole foods and nutritious meals being a limiting factor when it comes to T2D prevention among poorer populations. As highlighted by [2], to combat the daunting projections involving T2D, a united international strategy is required. A joint effort to provide nutrient-dense foods to vulnerable populations at low cost and programs to raise awareness about the benefits of exercise and mental well-being are necessary to reduce the incidence of not only T2D but of other metabolic syndrome diseases as well. 

In light of the global rise in cases of T2D, novel therapeutical approaches to help prevent and/or manage the disease are required. Additionally, the multifactorial nature of T2D and other metabolic diseases call for treatments that can interfere with multiple biochemical hallmarks and mechanisms in order to increase their success rates. In this regard, phenolic compounds may be strong candidates due to their multifunctional effects. This group of secondary plant metabolites are heavily concentrated in dietary sources associated with health promotion, such as fruits, vegetables, cereals, legumes, marine species, and nuts. Phenolics are believed to be important components for the risk reduction of cardiovascular diseases, some cancers, obesity, neurological ailments, and type 2 diabetes. This bioactive class of compounds is being increasingly recognized as important natural antioxidants, capable of restoring cellular redox homeostasis and preventing free radical-induced damage to lipids, proteins, and DNA [3]. 

In fact, the antioxidant properties of polyphenols, which can be exerted through multiple mechanisms, such as free radical scavenging and metal chelation, is of utmost importance for treating T2D. Insulin resistance, a major biological hallmark of this disease, is related to the increased levels of reactive oxygen species (ROS), leading to chronic oxidative stress and inflammation. In addition, excessive ROS can also result in the formation of oxidized LDL-cholesterol, an initial event leading to the generation of atherosclerotic plaques. Atherosclerosis and related cardiovascular diseases are among the possible complications of diabetes that may arise from poor management of the disease. Many scientific reports have documented polyphenolic action on the reduction of oxidative stress, insulin resistance, dyslipidemia, and inflammation, namely phenolic acids (e.g., chlorogenic acid), flavonoids (e.g., quercetin), proanthocyanidins (e.g., procyanidin B2), gallotannins (e.g., monogalloyl hexoside), and ellagitannins (e.g., ellagic acid hexoside). Moreover, glucose metabolism may be enhanced by, among other factors, the ability of phenolic compounds to serve as inhibitors of carbohydrate-digesting enzymes, such as α-amylase and α-glucosidase. In the gut, polyphenols are associated with the proliferation of beneficial microorganisms and restoring the integrity of the mucosal barrier, preventing dysbiosis which is also a relevant condition in diabetic patients [4,5].

The effectiveness of polyphenols in ameliorating diabetic biomarkers is highly dependent on their chemical structures and characteristics related to their digestion, absorption, and metabolism. These aspects greatly vary across dietary phenolic sources and have been addressed in a number of scientific studies using in vitro models [6,7], cell-based approaches [8,9], and in vivo experimentation [10,11]. Therefore, this review compiles selected up-to-date evidence about the effect of naturally sourced polyphenols on T2D and discusses their possible mechanisms of action. It also provides information on the required structural features for the management of this condition. 

## 2. Type 2 Diabetes 

**Figure 1:**
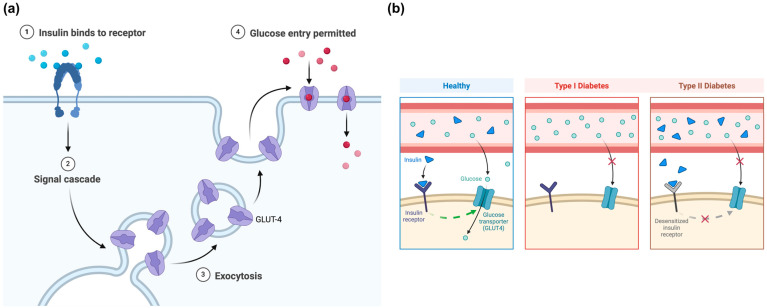
(**a**) Insulin pathway for glucose cellular absorption, and (**b**) difference between type 1 and type 2 diabetes. Created with Biorender.

Type 1 (T1D) and type 2 diabetes are the most predominant forms of diabetes, although other forms and subtypes also exist. For instance, latent autoimmune diabetes in adults (LADA) and maturity-onset diabetes of the young (MODY) are frequently misdiagnosed as type 2 or type 1 diabetes [2]. T2D is by far the most widespread diabetes type, as over 90% of diabetic individuals manifest this form of the disease. T1D is an autoimmune disease, characterized by the destruction of the β-cells in the pancreas, where insulin is produced. The hormone insulin is responsible for regulating the levels of blood glucose, being released in the bloodstream in response to rising glucose levels. Insulin promotes the uptake of glucose by the cells for use as an energy fuel by binding to insulin receptors in the cell membrane, activating signaling pathways that result in the translocation of glucose transporter type 4 (GLUT4) to the membrane’s surface (Figure 1a). GLUT4 facilitates the passive transport of glucose, which is oxidized to water and carbon dioxide, with the production of adenosine triphosphate (ATP). Alternatively, insulin also participates in the storage of excess glucose in the liver and muscles as glycogen, among other functions [12]. 

Upon the activation of T-cells, an autoimmune attack leads to the inflammation and destruction of β-cells, triggering the onset of T1D. As such, the amount of insulin available is progressively diminished until the insulin supply in the body is minimal or nonexistent. As a result, glucose is not absorbed, causing blood sugar levels to rise (hyperglycemia). Persistent hyperglycemia can damage several tissues and affect the normal functions of vital organs [13]. The complications of untreated diabetes include diabetic ketoacidosis (DKA), heart failure, neuropathy, retinopathy, joint and bone disorders, and skin conditions, among others. DKA is a consequence of glucose deprivation due to its insufficient cellular uptake. In this scenario, fat is used as an alternative energy source. By-products of fat breakdown include ketones and accumulate in the blood and urine, leading to metabolic acidosis. DKA is a life-threatening complication that can lead to coma and death if insulin therapy is not administered immediately. As T1D symptoms, such as excessive thirst and urination, rapid weight loss, extreme fatigue, and blurred vison, appear when 80–90% of β-cells have already been destroyed, it is not uncommon that a great proportion of patients are only diagnosed after the onset of DKA; however, untreated T2D can also lead to this state as well as all other complications [14]. Hyperglycemia is a common factor for both T1D and T2D. However, the mechanisms leading to this state significantly differs between the types of diabetes. In T2D, hyperglycemia is a result of increased insulin resistance. In fact, as the cells become less sensitive to insulin, β-cells try to compensate by producing a surplus of this hormone [2], marking a major difference between T2D and T1D, in which insulin is not synthesized (Figure 1b). 

The poor management of T2D results in a progressive decline of pancreatic β-cells, which can ultimately lead to ceasing insulin production. In this case, T2D patients become insulin-dependent, similar to T1D patients. On the contrary, an effective T2D control can lead to the restoration of β-cell function, which may occur by the recovery of previously inactive β-cells or by the redifferentiation of other pancreatic cells into new β-cells. In some cases, T2D can be reversed, especially in its early stages. Moreover, prediabetic patients (glycated hemoglobin A1c (HbA1c) of 5.7–6.4%) may avoid the onset of T2D by undergoing significant lifestyle changes, such as the adoption of a healthier diet, exercise, considerable weight loss, and the use of blood sugar-lowering medication, among other strategies [12].

T1D is believed to be dictated by a combination of genetic predisposition and environmental triggers, such as viral infections and toxins, although the exact causes of T1D remain to be clarified. This diabetes type commonly appears early in life during childhood and adolescence, although late-onset T1D can also occur. On the other hand, T2D is more common in adults over 55 years, albeit early T2D onset (≤40 years old) is becoming more frequent, being a consequence of poor dietary habits and a sedentary lifestyle. Notably, genetic predisposition can also play a factor in T2D risk. In essence, T2D is mostly preventable and even after its onset, reversal is possible when effective treatment is in place [13].

Over emphasis on hyperglycemia may sometime overshadow the multifactorial nature of T2D. In fact, β-cell failure can result from 12 pathophysiological defects. Besides direct β-cell defect, damage surfacing from the kidney, α-cells, pancreatic amylin, adipose tissue, skeletal muscles, liver, brain, gastrointestinal tract, inflammation, immune function, stomach, and small intestine may lead to T2D onset [12]. Therefore, the development of therapeutic approaches to treat this disease requires a holistic view of the body and opens several possibilities for treatments with distinct modes of action.

Importantly, mitochondrial dysfunction is a characteristic of T2D and other metabolic syndrome diseases, leading to insulin resistance. In the adipose tissue, mitochondrial dysfunction is linked with impaired adiponectin secretion, an insulin-sensitizing adipokine. In other tissues, insulin resistance is believed to be a consequence of increased levels of reactive oxygen species (ROS), which activates redox-sensitive serine kinases to phosphorylated insulin receptor substrate (IRS) proteins. However, the debate about whether mitochondrial dysfunction is a cause, or a consequence of insulin resistance is ongoing [15]. 

Insulin resistance can also surface from systemic inflammation, where increased levels of pro-inflammatory cytokines, such as interleukin-6 (IL-6) and tumor necrosis factor-alpha (TNF-α), cause the activation of downstream kinases, contributing to the phosphorylation of serine residues in IRS proteins. The infiltration of pro-inflammatory macrophages into the adipose tissue is also a factor that contributes to insulin resistance. Besides contributing to the onset of T2D, excessive ROS proliferation and systemic inflammation are also involved in the progression of diabetic complications, such as hypertension, myocardial infarction, and stroke, among others. As a multifactorial disease, T2D treatment can target multiple pathways, especially when considering drug administration. Metformin is the most administered antidiabetic medication in the world, targeting the reduction of blood glucose and HbA1c. Medication intended to increase insulin production is also available, including sulfonylureas. Nevertheless, both metformin and some types of sulfonylureas do not provide long-term protection to β-cell function and may even speed their decline, causing HbA1c to rise overtime. Some drugs target intestinal glucose absorption instead of focusing on insulin secretion. Medications such as acarbose and voglibose slow down carbohydrate digestion and absorption by an enzymatic inhibition process. Although these drugs are generally effective in preventing the conversion of prediabetes to T2D, their continuous administration is linked to gastrointestinal side effects, including diarrhea, abdominal pain, nausea, and vomiting [16,17]. The quest for improved quality of life for T2D patients motivates the procurement of alternative approaches to ameliorate diabetes symptoms. In this regard, naturally sourced bioactive molecules, such as polyphenols, are strong candidates. 

## 3. Bioactive Compounds

Eating a healthy diet is one of the most important strategies to prevent and manage T2D. The diet should be rich in whole foods and include quality nutrients, such as complete protein, fiber, and high-quality fats rich in omega-3 and omega-6 fatty acids. Micronutrients such as vitamins and minerals should also be present, as should phytochemicals. To achieve this, meals should be comprised of whole grains and cereals, fruits and vegetables, nuts, olive oil, fish, restricted red meat (preferentially lean), and dairy. In addition to containing all the aforementioned nutrients required for preventing and controlling T2D and other metabolic syndrome ailments, such foods are excellent sources of bioactive compounds. These substances are not classified as nutrients, but can have a manifold effect on human physiology, including ROS and inflammation reduction, inhibition of metabolic enzymes, amelioration of immune function, and overall oxidative protection to biomolecules. As such, bioactive compounds are believed to be the culprits behind the common association between healthy eating habits and the reduced risk of all-cause mortality [18]. The most commonly found bioactive groups are carotenoids, phytosterols, alkaloids, glucosinolates, tocopherols and tocotrienols, and phenolic compounds.

### 3.1. Phenolic Compounds 

Phenolic compounds are one of the most extensive and diverse group of bioactives in food and are synthesized in plants from precursor amino acids phenylalanine and tyrosine. They act as natural defenses against abiotic stress and are mostly procured for their antioxidant activity. This can be exerted through multiple mechanisms, including free radical scavenging, reducing power and metal chelation. As a heterogenous class of molecule, they are chemically characterized by at least one aromatic ring substituted by one (monophenols) or more hydroxyl groups (polyphenols). Most phenolic compounds are polyphenols and can be categorized according to their structural similarities [19].

- Flavonoids: C_6_-C_3_-C_6_ compounds characterized by two aromatic rings (A and B) connected to a central heterocyclic ring (C). Their pattern of hydroxyl and methyl substitutions define further classifications into flavonols (e.g., quercetin), flavones (e.g., luteolion), flavan-3-ols (e.g., catechin), flavanonols (e.g., dihydroquercetin), flavanones (e.g., naringenin), isoflavones (e.g., genistein), and anthocyanins (e.g., cyanidin). The latter subgroup comprises water-soluble pigments containing a sugar moiety. These pigments can be red, purple, or blue depending on the pH. Flavonoids are the phenolic class containing the highest number of compounds (over 8000 identified molecules).
- Hydroxycinnamic acids: Derivatives of cinnamic acid, with a C_6_-C_3_ structure. Examples include caffeic, *p*-coumaric, ferulic, sinapic, and chlorogenic acids. Most phenolic acids found in nature belong to this subgroup.
- Hydroxybenzoic acids: Derivatives of benzoic acid, with a C_6_-C_1_ structure, carrying at least one hydroxyl group. Examples include gallic acid and its dimeric form ellagic acid.
- Proanthocyanidins or condensed tannins: Tannins are oligomeric and polymeric phenolics that can be either condensed or hydrolysable. Condensed tannins (also known as proanthocyanidins) are formed by repeating units of catechin or epicatechin. Therefore, they can also be classified as flavan-3-ols, and include procyanidins ([epi]catechin polymers), prodelphinidins ([epi]gallocatechin polymers), and propelargonidins ([epi]afzelechin polymers).
- Hydrolysable tannins: Esters of gallic (gallotannins) and ellagic (ellagitannins) acids.
- Other phenolic groups include: lignans (derived from cinnamic acid derivatives), stilbenes (C_6_-C_2_-C_6_ compounds with a 1,2-diphenylethylene functional group, in which resveratrol is the most representative compound), coumarins (C_9_H_6_O_2_ compounds arranged in a bicyclic structure with lactone carbonyl groups), and hydroxytyrosol (a phenylethanoid derivative encountered in olive oil).

### 3.2. Antioxidant Function of Phenolic Compounds

The hydroxyl groups attached to the aromatic rings of polyphenols are essential for their functionality. These compounds can scavenge free radicals by hydrogen atom transfer (HAT) or single electron transfer (SET). In some cases, a combination of both mechanisms may occur. As phenolic hydroxyl groups are hydrogen donors that neutralize free radicals, molecules with multiple OH groups, such as proanthocyanidins, gallotannins, and ellagitannins, are expected to display stronger antiradical ability. Nevertheless, it is important that OH groups are located in regions with no steric hindrance for an effective transfer, as observed in phenolics carrying catechol and galloyl moieties. These particular structures can also act as secondary antioxidants by chelating transition metal ions, which are initiators of oxidative reactions [20]. 

In human physiology, polyphenols serve as exogenous antioxidants to counterbalance the excessive proliferation of peroxyl and hydroxyl radicals, which induce damage to vital biomolecules, such as lipids, proteins, and DNA. Phenolics are also believed to stimulate the production of endogenous antioxidant defenses, such as the enzymes catalase and superoxide dismutase (SOD). Oxidative damage is a common biological marker in several diseases, including atherosclerosis, type 2 diabetes, dementia, and some cancers [21]. Therefore, polyphenols may function as natural therapeutical aids for the prevention and management of such conditions, which will be further explored in Section 4 when discussing the role of polyphenols in T2D treatment.

### 3.3. Bioefficiency of Phenolic Compounds

The effectiveness of polyphenols as antioxidants and antidiabetic agents depends not only on their structural characteristics and the accessibility of their functional groups, but also on their digestibility, absorption, and metabolism in the human body. Polyphenols can exist in natural sources in three main forms—free molecules, conjugated with soluble components (e.g., sugars, organic acids), or insoluble-bound that link to structural macromolecules such as fiber, protein, and cellulose. The proportion of these different forms is determined by the food matrix. For instance, while fruits and vegetables predominantly carry phenolics in the free and soluble conjugate forms, cereals and legumes can have up to 60% of their total phenolics as insoluble-bound molecules. These proportions can vary within food groups and even among different fractions of the same material. For example, the seeds of certain fruits have a predominance of insoluble-bound phenolics, while the flesh is mainly composed of their soluble counterparts [22].

Importantly, free, soluble conjugates, and insoluble-bound phenolics show different digestibility patterns. Generally, free phenolics such as monomeric caffeic acid and quercetin are easily detached from the food matrix upon consumption and can reach the gastrointestinal tract, where they become available for small intestinal absorption. This characteristic is known as bioaccessibility, which is usually high for substances with a low molecular size and readily soluble in the digestive juices. Simple phenolic acids and flavonoids in the free state have been extensively reported as having high bioaccessibility and satisfactory ability to cross the intestinal membrane. Absorption in the small intestine commonly occurs via passive transport. However, some phenolic glycosides have been reported to use sodium-dependent glucose transporter (SGLT 1) for active transport or the glucose transporter 2 (GLUT 2) pathway for facilitated transport. Alternatively, phenolic glycosides may have their sugar moiety removed by lactase-phlorizin hydrolase (LPH) and undergo passive transport as aglycones [23]. 

Once absorbed, phenolics travel to the liver where they can undergo sulfation, glucuronidation, and methylation. In many cases, the metabolites produced show higher biological activity than the original phenolic molecules. The ability of polyphenols and other molecules to be absorbed, reach the systemic circulation, and be transferred to target tissues is known as bioavailability and can be a limiting factor for the utilization of phenolic compounds as therapeutic agents. It is estimated that only 5–20% of polyphenols in foods are bioefficient, meaning that they display satisfactory bioaccessibility and bioavailability [23]. 

Most phenolics, including soluble conjugates and insoluble-bound compounds, are unable to go through digestion, instead diverting to the colonic lumen, where microbial fermentation occurs. This is commonly the fate of more complex polyphenols, such as A-type and B-type procyanidins (high degree of polymerization), methyl gallate, and ellagic acid hexoside as their high-molecular size and often conjugation with other molecules hinder their ability to be absorbed in the small intestine. In the colonic route, the bonds between phenolics and other food components are broken and the fermentation of released aglycones can afford a variety of metabolites produced by demethylation, oxidation-reduction, decarboxylation, dihydroxylation, and isomerization reactions. A fraction of phenolic metabolites produced in the colon can then be absorbed into the enterocytes. Therefore, studies about the absorption and metabolism of phenolic compounds that do not include the colonic fermentation step may significantly underestimate their bioavailability rates [24].

In addition, the effect of polyphenols on the modulation of the gut microbiota is being increasingly recognized. In short, a healthy gut should contain a rich variety of microorganisms capable of stimulating the metabolism of dietary fiber into short-chain fatty acids (SCFAs). In turn, SCFAs play a role in maintaining the integrity of the intestinal barrier and are also involved in several inflammation-reducing mechanisms. In the colonic environment, phenolics can lead to the production of SCFAs and the reduction of dysbiosis, an imbalance in the composition of the gut microbiota, characterized by a poor variety of microorganisms where a high proliferation of pathogenic bacteria is observed, damaged intestinal barrier, and chronic inflammation. Dysbiosis can develop as a consequence of poor dietary habits and a sedentary lifestyle, being a biological marker of serious health issues such as inflammatory bowel disease, irritable bowel syndrome, and colorectal cancer [25]. As such, even when phenolics are not subjected to small intestinal absorption, they can still beneficially impact the gastrointestinal system and promote good health.

## 4. Polyphenols as Antidiabetic Agents 

Polyphenols have been demonstrated to ameliorate several risk markers of type 2 diabetes and its complications, such as improvement of insulin sensitivity, inhibition of α-amylase and α-glucosidase, reduction of inflammatory cytokines, prevention of dyslipidemia, oxidative stress reduction, and enhancement of endothelial function. Hence, they are strong candidates for replacing or augmenting the action of common diabetes medication. Evidence from in vitro and in vivo studies are discussed in subsequent sections.

### 4.1. In Vitro Evidence

Polyphenols may serve as natural inhibitors of α-amylase and α-glucosidase, while also reducing the formation of pro-inflammatory cytokines and damage to LDL-cholesterol (Table 1).

#### 4.1.1. Inhibition of α-Amylase and α-Glucosidase

The intake of rapidly digestible carbohydrates tends to cause postprandial blood sugar spikes. Starch, the most abundant polysaccharide in the standard diet, is found in several staple foods such as rice, potatoes, pasta, bread, and cereals. This polysaccharide exists in the form of granules composed of amylose and amylopectin. Amylose is characterized by linear chains of α-d-glucose linked by α-1,4 glycosidic bonds, while amylopectin has a branched structure where α-d-glucose units are joined by α-1,6 glycosidic bonds at the branch sites. Starch digestion begins in the mouth, where the enzyme α-amylase in saliva starts breaking down amylose, with the bulk of the process occurring in the stomach and small intestine due to pancreatic α-amylase. The breakdown of amylose results in α-dextrin and oligosaccharides such as maltose and maltotriose. These oligosaccharides are further broken down into glucose monomers for intestinal absorption by the action of intestinal α-glucosidase [31]. Therefore, the high activity rate of α-amylase and α-glucosidase intensifies postprandial hyperglycemia in diabetic patients, as glucose is released into the bloodstream without efficient cellular absorption due to insulin resistance. As such, several T2D treatments focus on suppressing the activities of metabolic enzymes involved in carbohydrate digestion to avoid postprandial hyperglycemia [32]. The starch type can influence the enzymatic digestion process, as not all starches are created equally. For instance, resistant starches are able to bypass digestion and move intact to the large intestine, similarly to dietary fiber. As such, they are not subjected to the action of α-amylase and α-glucosidase. These starches can be naturally encountered in potatoes, green bananas, and rice, among other sources, as well as produced by retrogradation or chemical modification. Resistant starches are positively linked to type 2 diabetes prevention by improving insulin sensitivity, lipid profile, and gut microbiota composition [33]. In an animal study [34], mice fed rice with a high content of resistant starch showed lower fasting glucose, total cholesterol, and LDL-cholesterol, while their levels of serum insulin and HDL-cholesterol were improved. The intervention was also positive in terms of gut microbiome composition, with enhanced production of short-chain fatty acids, followed by the upregulation of probiotic microorganisms and downregulation of harmful bacteria.

Standard diabetes drugs, such as acarbose, target key metabolic enzymes. However, the quality of life for T2D patients can be greatly impacted by the inhibition mechanisms used by these drugs. When inhibitors completely block enzymatic activity, undigested polysaccharides reach the colon, where intense microbial fermentation takes place. As a result, patients can experience abdominal discomfort, including pain, bloating, and diarrhea. To avoid these side effects, enzyme inhibitors should slow down carbohydrate digestion rather than stopping it altogether. This approach would reduce postprandial hyperglycemic events while preventing the presence of intact polysaccharides in the colonic environment. As such, polyphenols from natural sources, have been investigated as potential α-amylase and α-glucosidase inhibitors (Table 1) to aid in the management of T2D [35].

The ability of polyphenols to inhibit carbohydrate-digesting enzymes can be assessed in vitro by incubating the active substance (e.g., purified phenolics, phenolic extract) with the enzyme (e.g., α-amylase, α-glucosidase) and providing the substrate (e.g., starch, *p*-nitrophenyl-α-d-glucopyranoside). The reagents should be diluted in appropriate buffers, and the reaction is usually conducted under physiological conditions (pH 6.8, temperature 37 °C). A control experiment is generally carried out in parallel, containing all reagents without the active substance. The extent of enzymatic hydrolysis is measured spectrophotometrically by estimating the amount of product formed. The results can be expressed either as a percentage of inhibition, where the enzymatic activity in the presence of the inhibitor is compared to the enzymatic activity without any inhibition, or as half-maximal inhibitory concentration (IC_50_), which indicates the amount of inhibitor needed to reduce the enzymatic activity by 50%. The latter is often employed for isolated phenolics, where the assay involves testing multiple concentrations of the compound and is a measure of its efficacy as an inhibitor. Experiments on enzyme kinetics can also be performed to elucidate the inhibition mode of the phenolic substance by analyzing the changes in the maximum rate of reaction (V*max*) and Michaelis–Menten constant (K*m*) resulting from incubating the enzyme with the potential inhibitor [36].

The inhibition mode of polyphenols is what makes them strong therapeutic candidates for T2D control. Most polyphenols exhibit mixed inhibition mode toward α-amylase and α-glucosidase, i.e., the inhibitor can bind both the enzyme and the enzyme-substrate complex, altering the maximal reaction rate (V*max*) and the Michaelis–Menten constant (K*m*) of enzyme-catalyzed reactions. In this case, although the enzymatic activity is greatly reduced by the inhibitor presence, catalysis can still occur. Nevertheless, as phenolic compounds constitute a large heterogenous group of compounds, notable differences in the inhibition mode and efficacy of polyphenols can happen according to their structural particularities [37,38]. A study by Aleixandre et al. [39] examined the capacity of hydroxycinnamic and hydroxybenzoic acids to serve as α-amylase and α-glucosidase inhibitors. It was found that phenolic acids with several hydroxyl groups in their structure (e.g., chlorogenic, gallic, and protocatechuic acids) displayed high inhibitory activity toward both enzymes. This result is consistent with the essential role of hydroxyl groups for the phenolic–enzyme interaction. Generally, such interaction occurs via hydrogen bonding between the phenolics OH group and amino acids from the enzyme’s active site, although hydrophobic interaction and van der Walls forces have also been deemed relevant for phenolic–enzyme binding. Additionally, Yu et al. [27] highlighted the importance of salt bridges for the interaction between phenolic acids and α-glucosidase, which was demonstrated by molecular docking analysis and preferentially occurred between the carbonyl group on cinnamic, 3,4-dimethoxycinnamic, caffeic, and ferulic acids and the Arg 329 residue in the active site of the enzyme.

According to Zhu et al. [40], two distinct binding modes between flavonoids and enzymes are possible, including flavonoids directly attaching to amino acid residues at the enzyme’s active sites, thereby preventing substrate binding; as well as interaction between flavonoids and amino acid residues adjacent to the active site, obstructing access to the active center. The authors also observed that the presence of caffeoyl, galloyl, and prenyl moieties in flavonoids can enhance their inhibitory activities. The galloyl moiety is also an enhancing feature for the enzymatic inhibition capacity of gallotannins. Moreover, steric hindrance is believed to reduce the inhibitory efficacy of flavonoids, with linear molecules generally being more potent inhibitors of porcine pancreatic α-amylase. In fact, the exposure of OH groups is key for flavonoids to achieve high α-amylase inhibitory activity. Flavonoid structures where the OH group is positioned at C5, C6, and C7 on the A ring, as well as at C4 on the B ring, are more favorable for enzymatic binding. Similarly, the conjugation of the 4-carbonyl with 2,3-double bonds enhances electron delocalization between the A and C rings, stabilizing π-π stacking between flavonoid aromatic rings and tryptophan’s indole ring at the active site of α-amylase [41].

A phenolic-rich extract from guarana powder consisting of the insoluble-bound fraction showed a dose-dependent capacity to inhibit α-glucosidase, reaching a half-maximal inhibitory dose (IC_50_) of 1.624 μg/mL whereas the positive control used in the experiment (acarbose) showed IC_50_ values ranging from 36.0 to 107.3 μg/mL [37]. Moreover, the extract inhibited the enzyme by mixed inhibition mode, confirming the trend seen in other publications. An analysis of the profile of the insoluble-bound phenolic extract from guarana revealed a great variety of proanthocyanidin dimers, trimers, and tetramers. These high molecular weight compounds show an extensive degree of hydroxylation, offering a number of interaction sites for the phenolic-enzyme binding, which may explain the extract’s high affinity for α-glucosidase and consequent strong inhibition power [37]. 

The enzymatic inhibitory action of polyphenols is highly dependent on their molecular structures. Therefore, it is reasonable to assume that structural changes induced by the gastrointestinal digestion process can significantly impact the ability of phenolics to inhibit enzymes such as α-amylase and α-glucosidase. These changes may either enhance or decrease this bioactivity, depending on whether the alterations favor the formation of metabolites with the necessary structural apparatus to optimize phenolic-enzyme interactions. Due and Myracle [42] conducted in vitro simulated gastrointestinal digestion of both unfermented and fermented aronia kefir, reporting alterations in the phenolic profile following gastric and intestinal digestion. The results showed that under gastric conditions, there was a high release of anthocyanins and phenolic acids. Anthocyanins remained predominant after small intestine digestion, along with chlorogenic acid. This phenolic profile resulted in a strong inhibitory capacity of digested kefir toward α-glucosidase, particularly in fermented samples (IC_50_ of 152.53 mg/mL compared to 365.16 mg/mL in unfermented samples). α-Amylase was also inhibited by intestinally digested kefir, although the effect of fermentation was less pronounced (IC_50_ of 146.52 mg/mL compared to 196.21 mg/mL in unfermented samples). In another study, chlorogenic acid was also reported as a strong α-glucosidase inhibitor due to its high phenolic-enzyme affinity [43].

#### 4.1.2. Suppression of Pro-Inflammatory Cytokines

The intense propagation of reactive oxygen species (ROS) associated with insulin resistance is a triggering event leading to cellular damage and activation of inflammatory pathways. Oxidative stress alters the molecular structure and disrupts the normal biological functions of lipids, proteins, and DNA. This alteration prompts an inflammatory response that may result in a continuous state of chronic inflammation. Understanding key inflammation pathways can help develop novel therapeutical approaches to inhibit these mechanisms [44].

ROS activate the nuclear factor kappa-light-chain-enhancer of activated B cells (NF-κB), a transcription factor that plays a vital role in inflammatory processes. The activation mechanisms promoted by ROS include the phosphorylation and degradation of IκB, a NF-κB inhibitor, removing a barrier to the entrance of the transcription factor to the cell nucleus. In the nucleus, NF-κB prompts the transcription of a myriad of pro-inflammatory genes, which includes cytokines, chemokines, and adhesion molecules. Pro-inflammatory genes can also be expressed through mitogen-activated protein kinases (MAPKs), such as ERK, JNK, and p38, which activate transcription factors such as AP-1. Relevant pro-inflammatory cytokines/chemokines include interleukin-8 (IL-8), interleukin-6 (IL-6), and tumor necrosis factor-alpha (TNF-α). IL-8 is a chemokine stemming from NF-κB and MAPKs activation. It recruits neutrophils to the inflammation site, perpetuating the inflammatory response and potentiating tissue damage. The NF-κB pathway also produces IL-6, which carries out several roles in inflammatory cascades, such as the promotion of T cells and B cells differentiation, as well as the stimulation of acute-phase proteins production by the liver. Following NF-κB activation, macrophages, T cells, and other cells produce the cytokine TNF-α, which induces the production of other pro-inflammatory cytokines, intensifying immune cells recruitment and potentiating vascular permeability [45].

ROS-triggered inflammatory pathways can also produce cyclooxygenase-2 (COX-2), an enzyme that catalyzes the conversion of arachidonic acid to prostaglandins, lipophilic substances that advance inflammation, pain, and fever [46]. In addition, T cells and natural killer cells generate interferon gamma (IFN-γ), which activates macrophages and leads to the production of pro-inflammatory cytokines. Besides, IFN-γ upregulates the expression of major histocompatibility complex (MHC) molecules, strengthening antigen presentation and the adaptive immune response. The activation of inflammatory pathways and constant production of pro-inflammatory cytokines and chemokines establish a feedforward mechanism with ROS, aggravating insulin resistance and contributing to the development of diabetic complications, such as arthritis, cardiovascular diseases, and neurological disorders [47]. Moreover, when the gastrointestinal tract is under a persistent state of oxidative stress, the intestinal mucosa is damaged by ROS action, intensifying the immune response and ensuing chronic inflammation. Inflammatory bowel disease, Crohn’s disease, and ulcerative colitis are conditions that can result from a chronically inflamed GI tract [48].

Phenolic compounds such as kaempferol, quercetin, apigenin, chrysin, luteolin, biochanin A, genistein, (−)-epigallocatechin-3-gallate (EGCG), butin, paenol, ellagic acid, and resveratrol have been reported to inhibit the activation of NFκB and MAPKs pathways and consequently reduce the production of IL-8, IL-6, TNF-α, IFN-γ, and COX-2. The in vitro investigation of phenolic-induced anti-inflammatory effects has been carried out in a number of cell-based studies, where macrophages (e.g., RAW 264.7), human monocytic cells (e.g., THP-1), and endothelial cells (e.g., HUVECs) have been employed [8,9,49]. Generally, the experiments involve the incubation of the aforementioned cells with pro-inflammatory stimuli (e.g., lipopolysaccharide (LPS), TNF-α, IL-1β) to induce the formation of pro-inflammatory cytokines. Treatment cells are also incubated with the phenolic substance in multiple concentrations and compared with a negative control only containing the pro-inflammatory agents. To assess whether phenolics are capable of mitigating the production of cytokines, enzyme-linked immunosorbent assay (ELISA), multiplex cytokine assays, or quantitative PCR (qPCR) can be used. Iin addition, the inflammatory mechanisms can be analyzed by investigating the expression of inflammation genes and proteins through Western blotting, qPCR, or immunofluorescence [50]. 

Zhang et al. [51] obtained an anthocyanin-rich extract from purple carrot and potato, further applying it to human intestinal epithelial (Caco-2) cells to evaluate its anti-inflammatory effects. A Caco-2 monolayer was prepared and mixed with an inflammatory agent (hydrogen peroxide) to induce the formation of pro-inflammatory cytokines, namely (IL)-1β, IL-6, IL-8, and TNF-α. Extracts were tested at 5, 10, 50, and 100 μg/mL. The results showed that extracts obtained from purple carrot and potato are predominantly composed of cyanidin and petunidin aglycones and are particularly effective at reducing the formation of all pro-inflammatory cytokines measured, especially when applied at 50 and 100 μg/mL. The higher concentrations were also capable of inducing elevated levels of endogenous antioxidant enzymes, such as catalase, superoxide dismutase (SOD), glutathione peroxidase, and glutathione reductase. For instance, the purple carrot extract elevated SOD levels in 61.8 and 68.6% at 50 and 100 μg/mL, respectively. Similarly, the same enzyme was elevated in 68.7 and 79.6% when purple potato extract was incubated with Caco-2 cells at 50 and 100 μg/mL, respectively.

A Caco-2 monolayer has also been employed as an inflamed cellular model in another study [52] to assess the anti-inflammatory properties of multiple polyphenols. Purified resveratrol, ellagic and ferulic acids, curcumin, quercetin, chrysin, EGCG, and genistein were tested at 50 μM, which is consistent with expected phenolic concentrations in the intestinal epithelium. The results showed that genistein was able to decrease in 50% the production of IL-6 and monocyte chemoattractant protein-1 (MCP-1) compared with the control cells (no antioxidant treatment). The same was observed for EGCG, which also reduced the expression of IL-8 in 60%, confirming its efficacy in inflamed cells [52].

Berries are among the most popular sources of natural antioxidants, including polyphenols. Hence, Gu et al. [53] isolated the phenolic fractions from cranberries, black raspberries, red raspberries, strawberries, blueberries, and blackberries, with further incubation with LPS-stimulated RAW264.7 macrophages. Control macrophages (no berry phenolics) showed an excessive expression of nitric oxide, prostaglandin E2 (PGE2), COX-2, IL-6, and TNF-α. All these inflammatory parameters were drastically reduced by the application of berry phenolics, which in selected instances were able to achieve up to 94% reduction in the production of pro-inflammatory agents. Moreover, berry phenolics blocked the phosphorylation of p65 and IκBα degradation, which impacted on the downregulation of the NF-κB pathway.

#### 4.1.3. Mitigation of LDL-Cholesterol Oxidative Damage

Cholesterol is an integral component of biological membranes, regulating their fluidity and ensuring their integrity upon temperature fluctuations. Additionally, cholesterol is a precursor of steroid hormones, vitamin D, and bile acids [54]. Cholesterol is transported through the bloodstream by lipoproteins, primarily high-density lipoprotein (HDL) and low-density lipoprotein (LDL). Lipoproteins are heterogeneous molecules composed of lipids and proteins. While LDL carries cholesterol from the liver to the tissues, HDL removes cholesterol from the bloodstream and transports it back to the liver for excretion. This reverse transport performed by HDL is crucial for preventing plaque buildup in the arteries and is why HDL is referred to as “good cholesterol”. Meanwhile, LDL contributes to the deposition of cholesterol in the arteries and further clot formation, narrowing blood vessels and potentially leading to the onset of cardiovascular diseases. Ideally, LDL should be maintained at low levels while HDL concentration should be high to ensure good cardiovascular health. Additionally, excessive levels of circulating LDL increase the chances of accumulation of its oxidized species (Ox-LDL). Oxidized LDL is highly composed of adherent molecules, which facilitates its deposition in the endothelium and the inner lining of blood vessels. The presence of Ox-LDL activates an inflammatory cascade that results in the recruitment of macrophages to uptake highly oxidized LDL. This event leads to the formation of dysfunctional macrophage foam cells that accumulate on the blood vessels, further establishing atherosclerotic plaques. The progression of atherosclerosis causes blood flow restriction and can result in stroke and myocardial infarction [55].

Balancing LDL-c and HDL-c is central for diabetes management in order to prevent cardiovascular complications, as insulin resistance is a risk factor for dyslipidemia. In the adipose tissue, insulin resistance hampers the regulation of hormone-sensitive lipase, leading to uncontrolled lipolysis of triacylglycerols. This causes an increase in the levels of circulating non-esterified fatty acids. In the liver, an excessive formation of triacylglycerol-rich lipoproteins occurs as insulin can no longer downregulate their production. These factors contribute to a dysfunctional lipid profile, with elevated triacylglycerols and LDL-c levels and insufficient concentration of HDL-c [56]. 

Polyphenols can protect the integrity of LDL-c due to their antioxidant activity, offsetting the formation of Ox-LDL and subsequent atherosclerotic plaque deposition. Phenolics are able to scavenge free radicals, safeguarding the integrity of lipids and proteins contained in the lipoprotein molecule. Alternatively, they can also complex with transition metal ions to arrest their prooxidant action. Such a mechanism is largely evaluated in studies assessing the cardioprotective effect of phenolic compounds. The experimental approach employed in this type of research usually involves the incubation of human LDL-c with a prooxidant agent (e.g., copper sulfate) at 37 °C, along with the studied antioxidant. Samples can be incubated for up to 22–24 h, with constant monitoring of oxidative product formation in order to establish the efficacy of the antioxidant in hampering the production of Ox-LDL. Conjugated dienes are common hallmarks of LDL-c oxidation. They result from the rearrangement of double bonds in polyunsaturated fatty acids, being a primary product of lipid peroxidation and occurring shortly after chain reaction initiation [57,58]. 

Conjugated dienes can be detected spectrophotometrically at 234 nm and are useful for estimating the oxidative stability of polyunsaturated fatty acids in the LDL-c molecule. By comparing test samples with a negative control (no exogenous antioxidants in the reaction mixture), it is possible to assess the efficacy of polyphenols as potential anti-atherosclerotic agents. In this assay, the initial hours of incubation generally produce a negligible amount of conjugated dienes, which can be due to the action of endogenous antioxidants contained in LDL-c, such as ubiquinol-10, β-carotene, and α-tocopherol. However, as time progresses, these natural defenses are progressively depleted. At this point, added phenolic compounds begin to exert their oxidative protection toward LDL-c [57,58].

The metal chelating property of polyphenols is favored by specific structural features, meaning that not all phenolics carry such ability; compounds bearing a catechol (adjacent hydroxyl groups at C3 and C4 on the B ring of flavonoids) and/or galloyl moiety are particularly efficient at serving as chelators of transition metal ions. The two adjacent OH groups of the catechol moiety can donate electron pairs to form coordinate bonds with vacant electron-accepting d-orbitals of metal ions. The same is observed for the three hydroxyl groups of the galloyl moiety, creating a stable metal–phenolic complex that serves to reduce the availability of metals for participating in redox reactions that lead to the production of highly reactive free radicals. (−)-Epigallocatechin gallate (EGCG) and (−)-epicatechin gallate (ECG) possess both catechol and galloyl moieties, making them great agents for LDL-c protection. Apart from their chemical structures, ECG and EGCG are also favored by their lipophilic character, facilitating their dissolution in LDL-c for performing their antioxidant effect [59].

The oxidative stress promoted by hyperglycemia leads to the formation of glycated LDL-c, which is highly susceptible to uptake by macrophages and consequent atherosclerotic plaque generation. However, Wu et al. [60] incubated human plasma with several concentrations (25–100 μM) of EGCG for 3 h, after which LDL-c was isolated from plasma and induced to oxidation by applying high-glucose CuSO_4_. The production of conjugated dienes and thiobarbituric acid reactive substances (TBARS) was monitored. The pre-incubation of EGCG with LDL-c provided the lipoprotein with a higher resistance to oxidation, acting in synergism with α-tocopherol, which was less depleted in the presence of EGCG when compared with the negative control. EGCG also reduced high glucose-mediated long-term glycation of LDL-c.

### 4.2. In Vivo Evidence

Polyphenols may have a positive effect on insulin sensitivity, lipid profile, and inflammatory biomarkers, as evidenced by several in vivo studies (Table 2).

#### 4.2.1. Effect of Polyphenols on Insulin Sensitivity

Insulin resistance is characterized by restricted insulin responsiveness in target tissues, causing the β-cells in the pancreas to overproduce the hormone. Excessive insulin leads to a progressive malfunction of the β-cells through oxidative stress. As such, type 2 diabetes is a consequence of hyperglycemia resulting from impaired glucose uptake, along with hyperinsulinemia and chronic inflammation. Obesity is one of the major risk factors for the onset of type 2 diabetes, with chronic inflammation assuming a critical role in the interplay between these two conditions. In obesity, nuclear factor kappa-light-chain-enhancer of activated B cells (NFκB) drives the secretion of pro-inflammatory cytokines (e.g., TNF-α, IL-6) by adipocytes. Consequently, macrophages are recruited and promote a feed-forward signaling mechanism by producing additional cytokines, intensifying inflammation and further increasing insulin resistance [70]. 

β-Cells cannot rely on endogenous antioxidants defenses and are extremely susceptible to ROS attack. Therefore, reversing T2D is more easily achieved during the initial stages of the disease. As ROS starts to proliferate uncontrollably and chronic inflammation ensues, β-cells are progressively damaged in an irreversible way, ultimately halting insulin production. However, therapeutic approaches are more effective when taken in the pre-diabetic stage or even as a preventive measure for individuals with increased T2D risk [71].

Insulin-secreting cell lines are widely used to study the impact of polyphenols on insulin responsiveness. Commonly, INS-1 β-cells from rat pancreas are employed and represent a suitable strategy for providing insights into polyphenolic mechanisms of action. Among the reported mechanisms through which phenolics can reduce insulin resistance, it is possible to mention the activation of signal-regulated kinase (ERK)1/2, Ca^2+^ influx, reduction of nuclear Foxo1 localization, and reduction of akt phosphorylation. Youl et al. [72] demonstrated that quercetin at 20 μmol/L was able to enhance glucose and glibenclamide-induced insulin secretion in INS-1 β-cells, while also augmenting the activation of the ERK1/2 pathway, involved in the regulation of glucose-induced insulin secretion in β-cells. The same study showed that quercetin could also protect β-cells from oxidative damage caused by hydrogen peroxide (50 μmol/L). 

The ERK1/2 pathway starts when glucose is taken up by GLUT-2 in the β-cells. Once absorbed, glycolysis proceeds and glucose is oxidized with the formation of ATP. Thus, following glucose uptake, a high ratio of ATP/ADP is reached, reflecting on the closure of the K^ATP^ channel mediated by sulfonylurea receptor-1 (SUR-1). This closure leads to depolarization of the membrane potential, which is sensed by voltage-dependent calcium channels (VDCC), rising the intracellular levels of Ca^2+^ followed by the secretion of insulin granules. Calcium also increases cyclic adenosine monophosphate (cAMP) levels, resulting in the phosphorylation of ERK1/2 through the activation of protein kinase A (PKA). The activated form of ERK1/2 partakes on glucose-mediated remodeling of F-actin, resulting in insulin release from β-cells [73]. This mechanism has been demonstrated in INS-1 cells and rat pancreatic islets, with vanillic acid potentiating cAMP production and activation of PKA, leading to phosphorylation of ERK1/2. Moreover, the oral administration of 30 and 60 mg/kg of vanillic acid to male Winstar hyperglycemic rats showed increased serum insulin levels, with better results observed for the higher dose [73].

In another animal study [74], apple polyphenols (150 mg/kg of body weight) were investigated for their capacity to lower insulin resistance in 15-week-old obese Zucker rats. Polyphenols were administered in the form of an extract composed of phloridzin, chlorogenic acid, and quercetin. The polyphenolic treatment showed better postprandial glycemic regulation when compared to the control group, also demonstrating lower glucose and insulin levels after 4 weeks. According to the authors, the effect was due to a better ability of the peripheral tissues to respond to glucose uptake upon insulin stimulation promoted by apple polyphenols, which was achieved by GLUT4 translocation to the plasma membrane.

Polyphenols have also been implicated in interfering with incretin hormones, such as glucagon-like peptide-1 (GLP-1). This hormone promotes insulin release induced by postprandial glucose levels, when it is released by L cells in the distal small intestine and colon. In a human intervention study, participants (48 healthy adults) were given cacao polyphenol-rich chocolate (50 g), and their GLP-1 levels were analyzed. Results showed that subjects who consumed the chocolate demonstrated lower plasma glucose concentrations after 120 min following intake when compared to the control group. This observation was attributed to the early release of GLP-1 and serum insulin caused by the consumption of polyphenol-rich products [75].

Epicatechin and procyanidins are predominant polyphenols in cacao but are also present in several other foods. Berries, such as cranberry, blueberry, mulberry, and hawthorn berry contain a myriad of simple and polymeric flavonoids capable of modulating biomarkers associated with the onset of diabetes. For instance, a polyphenolic extract from hawthorn berry was able to regulate insulin resistance in skeletal muscle and liver of type 2 diabetic rats. The extract was mainly composed of procyanidin B2, epicatechin, chlorogenic acid, as well as dimeric, trimeric, and tetrameric procyanidins. Among the observed effects, the extract upregulated the SIRT1/AMPK/NF-kB pathway. SIRT1 from the mammalian sirtuins family is a NAD+ dependent protein deacetylase that can interfere with the transcriptional activation of NF-κB, inhibiting the NF-κB-mediated inflammatory pathway. Levels of SIRT1 protein in the liver were increased by 72%, while the expression of pro-inflammatory cytokines IL-6 and TNF-α was downregulated by 38 and 43%, respectively. Insulin resistance was also greatly improved by the administration of hawthorn berry extract, showing an effect similar to that of metformin [76].

#### 4.2.2. Effect of Polyphenols on Dyslipidemia

Polyphenols may attenuate lipid disorders by direct or indirect action. The former include suppressing the formation of Ox-LDL and by acting synergistically to restore endogenous antioxidant defenses. Meanwhile, polyphenols can work indirectly by stimulating the production of endogenous defenses through upregulation of the Keap-1/Nrf2 antioxidant defense system pathway, as well as preventing mitochondrial dysfunction through stimulation of the AMPK/SIRT1/PGC-1α pathway [77]. The Keap-1/Nrf2 pathway is responsible for expressing several antioxidant enzymes such as superoxide dismutase (SOD), glutathione peroxidase (GSH-Px), heme oxygenase-1 (HO-1), glutathione reductase, thioredoxin reductase, ferritin, and NAD(P)H:quinone oxidoreductase 1 (NQO1) [78]. In parallel, SIRT1 activation is prompted when energetic stress leads to an increase in the NAD^+^/NADH ratio. This event results in the deacetylation of PGC-1α by SIRT1, with consequent production of antioxidant proteins. AMPK modulates this process and secures mitochondrial homeostasis, which underlies the importance of the AMPK/SIRT1/PGC-1α network [79]. 

An animal study by Abdel-Moneim et al. [64] demonstrated that the administration of gallic and *p*-coumaric acids to diabetic rats greatly reduced their cardiovascular risk index 1 from 5.13 (control) to 2.42 (gallic acid treatment) and 2.89 (*p*-coumaric acid treatment), cardiovascular risk index 2 from 2.73 (control) to 0.99 (gallic acid treatment) and 1.29 (*p*-coumaric acid treatment), while raising the antiatherogenic index from 24.84 (control) to 81.08 (gallic acid treatment) and 54.58 (*p*-coumaric acid treatment). These effects were accompanied by improved levels of HDL-cholesterol.

Aslam et al. [65] administered *Opuntia fícus indica* fruit extract to atherosclerotic Winstar rats fed a high-fat diet (n = 40). The extract was mainly composed of quercetin, gallic, vanillic, and chlorogenic acids and two doses were tested (600 and 800 mg/kg/day). The treatments greatly improved the antioxidant status of the animals, with the downregulation of dual oxidases (Duox, Duoxa-1, and Duox-2 genes) expression. Dual oxidases contribute to the generation of free radical species, such as hydrogen peroxide, by transferring electrons from NADPH to oxygen. At the same time, the extracts were able to induce the upregulation of the Nrf2 pathway, leading to increased expression of antioxidant enzymes. These factors reflected on an improvement of the rats’ lipid profiles, with the highest concentration (800 mg/kg/day) being more efficacious in reducing hepatic lipid accumulation.

According to Hybertron et al. [80], a polyphenol-based dietary supplement (PB123), which is a combination of ginger root and rosemary extracts, as well as luteolin from *Saphora japonica*, was able to downregulate cholesterol biosynthesis in hepatocarcinoma (HepG2) cells by inhibiting the expression of 15 genes involved in this pathway. Consequently, lower levels of total cholesterol were registered in treated cells. Moreover, Nrf2 activation occurred in a dose-dependent manner (5–12 μg/mL), with the same being observed for the downregulation of liver fatty acid binding protein (FABP1). This protein has a high affinity for fatty acids, contributing to their uptake by the hepatic cells; therefore, inhibiting FABP1 may improve the lipid profile.

Sarriá et al. [66] conducted two parallel clinical trials to study the bioavailability and impact on cardiovascular disease risk factors of several cocoa products, including unprocessed raw cocoa powder and soluble cocoa products (dietary fiber-enriched, methylxanthines-enriched, conventional cocoa low in sugar, and phenolic-enriched). For bioavailability assessment, a crossover single-blind study was carried out and included 13 healthy participants (men and women) aged between 18 and 45 years. The flavonoids epicatechin and procyanidin B1 were predominant in the tested cocoa products. After their intake, 10 phenolic metabolites were detected in the plasma and 30 metabolites were found in the urine of study subjects. Among those, phase II derivatives of epicatechin were highly concentrated in the plasma from one to two hours after the intake, also representing 33% of urine metabolites. Meanwhile, 67% of urine metabolites were phase II derivatives of hydroxyphenyl-γ-valerolatones and valeric acid, formed in the colon upon biotransformation by the gut microbiota. For assessing the risk of cardiovascular disease, a randomized crossover study was conducted with male and female subjects ranging from 18 to 55 years old and classified as healthy and moderately hypercholesterolemic. The results showed that the consumption of the dietary fiber-enriched cocoa product for four weeks led to a considerable increase in the levels of HDL-c, while decreasing glucose and IL-1β (pro-inflammatory cytokine) concentration. Similarly, consuming the phenolic-enriched cocoa product for the same length of time resulted in increased HDL-c and no further changes in other biomarkers of cardiovascular diseases.

#### 4.2.3. Reduction of Inflammatory Processes

Type 2 diabetes is often reduced to its primary characteristics, that is insulin resistance and impaired glucose metabolism. However, this type of generalization oversimplifies the true complexity of T2D and lacks an understanding of the compromised metabolic system of diabetic patients. A holistic perspective about T2D can lead to the development of better therapeutical approaches to manage and treat the disease. Ideally, substances capable of ameliorating several T2D hallmarks are the gold standards for cutting-edge diabetic treatments. Case in point, Yang et al. [67] reported that the polyphenolic extract of *Phellinus baumii*, a wild fungus cultivated in China and used in folk medicine, could enhance inflammatory markers, improve insulin sensitivity, and reduce dyslipidemia in diabetic mice. The extract is mainly composed of osmudacetone, hispidin, davallialactone, 2,5-bis(4,7-dihydroxy-8-methyl-2-oxo-2H-chromen-3-yl) cyclohexa-2,5-diene-1,4-dione, hypholomin B, and inoscavin A [81]. In the 60-day study [67], 60 ICR male mice were divided into groups that included a negative control (no treatment), a positive control (100 mg/kg of body weight of metformin), and treatment groups receiving the phenolic extract (50, 100, or 150 mg/kg of body weight). The administration of the highest extract dose significantly improved insulin sensitivity index, comparable to that of the metformin group. The extract also improved glucose metabolism, measured by the levels of glucagon, epinephrine, and glycosylated hemoglobin, and reduced the levels of total cholesterol and LDL-cholesterol, particularly when the highest extract dose was administered (150 mg/kg). The incubation of the phenolic extract with RAW264.7 macrophages (LPS-induced inflammation) showed a dose-dependent decline in the production of the pro-inflammatory cytokines IL-6 and TNF-α. According to the authors, *Phellinus baumii* phenolics act by activating the IRS1/PI3K/AKT pathway [67]. 

Vinegar polyphenols were also reported to exert multiple functions to offset T2M biomarkers. According to Xia et al. [81], a vinegar phenolic extract composed of 29 polyphenols (*p*-hydroxybenzoic acid, ferulic acid, and ethyl ferulate were the main compounds identified) administered to diabetic mice fed a high-fat diet for 6 weeks led to a reduction in the levels of blood glucose, total cholesterol, and LDL-c, as well as an uptick in HDL-c levels, particularly when a high extract dose was employed (750 mg/kg of bodyweight). The extract was also reported to inhibit the TLR4/NF-κB signaling pathway, with consequent lower production of pro-inflammatory cytokines. In addition, vinegar polyphenols restored a healthy gut microbiota in diabetic mice by upregulating probiotic bacteria (*Lactobacillus, Bifidobacterium,* and *Bacteroides*) and downregulating harmful microorganisms (Firmicutes, Proteobacteria, and *Enterorhabdus*), while also enhancing the concentration of short-chain fatty acids.

A combined approach has been proposed by Mansour et al. [68], suggesting the co-administration of metformin and olive leaf phenolic extract (100 mg of each) to ameliorate key biomarkers associated with T2D. Polyphenols identified in olive leaf include oleuropein, salicylic acid, rutin, and *p*-hydroxybenzoic acid. The integrate action of these multiple phenolic compounds resulted in high α-glucosidase inhibitory rates, ranging from 85.54 to 92.61%. Besides, diabetic rats receiving the metformin–phenolic treatment were able to reduce their HbA1c from 13.80 (no treatment) to 4.70%. There was also a noticeable change in the lipid profile of treated animals, with the restoration of normal levels of total cholesterol, HDL-c, and LDL-c, followed by an improvement of their liver function. Similar effects were reported by Sarkar et al. [69] upon the administration of a phenolic extract from *Parkia javanica* edible pods to Swiss albino mice. The treatment decreased their average blood glucose from 462.66 to 228.66 mg/dL and also resulted in the upregulation of the antioxidant enzymes catalase and superoxide dismutase, as well as a decrease in malondialdehyde (a biomarker of lipid peroxidation) levels in liver and kidney tissues. 

## 5. Conclusions

Polyphenols can play a critical role in the prevention and management of type 2 diabetes, a metabolic disease with an alarming rise in prevalence, particularly in low- and middle-income countries. T2D is characterized by its multifactorial nature, which is influenced by lifestyle choices, genetic predisposition, and environmental factors. The evidence presented supports the notion that a diet rich in whole foods, particularly those high in phenolic compounds, can significantly mitigate the risks associated with T2D. These compounds exhibit potent antioxidant properties, which are essential in combating oxidative stress and inflammation, key contributors to insulin resistance and other metabolic disorders. Various classes of phenolic compounds can be sourced from natural products, carrying distinct mechanisms of action, which reinforces the idea that not all phenolics are created equal; their effectiveness is closely tied to their chemical structure and bioavailability. In short, the integration of phenolic-rich foods into dietary strategies represents a promising avenue for T2D management and prevention. In fact, the commercialization of polyphenol-based nutraceuticals aiming to reduce the incidence of T2D, as well as ameliorate its symptoms is already a reality, both in the form of isolated compounds (e.g., resveratrol, gallic acid, ellagic acid capsules) or as plant-source phenolic extracts (e.g., green tea extract, berry extract powder/capsules). However, claiming health effects on nutraceutical labels is still a regulatory grey area due to the lack of reproducible studies showing the exact mechanisms of polyphenols on T2D. Therefore, future research should focus on elucidating the specific pathways through which these compounds exert their beneficial effects, as well as exploring the potential for developing targeted therapeutic interventions. A holistic approach that combines dietary modifications, increased physical activity, and public health initiatives is essential to alleviate the burden of T2D and improve health outcomes for affected individuals. By fostering a deeper understanding of the interplay between diet and T2D, it is possible to pave the way for innovative solutions that address this pressing global health challenge.

## Figures and Tables

**Table 1:** In vitro studies reporting the effects of polyphenols in ameliorating diabetic hallmarks.

Polyphenols	Source	Bioactivity	Model	Mechanism of Action	Reference
Tannic acid, catechin, gallic acid, quercetin, epicatechin	-	Inhibition of α-amylase and α-glucosidase	In silico molecular docking	Hydrogen bonding between OH-phenolic groups and amino acid residues in the active site of enzymes	[26]
Ethanolic extract containing gallocatechin, epicatechin, procyanidin B, and ellagic acid, among others	*Lepisanthes fruticosa*	Inhibition of α-amylase and α-glucosidase	Enzymatic activity inhibition assays	Not evaluated	[6]
Cinnamic, 3,4-dimethoxy cinnamic, caffeic, and ferulic acids	Purified phenolic acids	Inhibition of α-amylase and α-glucosidase activity, reducing rapidly digested starch content, increasing resistant starch content	Enzymatic activity inhibition assays	π–π stacking interactions with α-amylase, salt-bridge interactions with α-glucosidase, stabilization by hydroxyl (OH) and methoxy groups on the benzene ring	[27]
Phenolic extract (composition not reported)	Rice bran	Anti-inflammatory properties	RAW264.7 mouse macrophage cells	Decrease in oxidative stress biomarkers (MDA, intracellular reactive oxygen species), reduction of nitric oxide, and pro-inflammatory cytokines (IL-6, IL-12p70, IFN-γ) production, via metal chelating properties and free radical scavenging activity	[8]
Quercetin	Purified flavonoid	Modulation of endothelial cell metabolism, anti-inflammatory effects	Human umbilical vein endothelial cells (HUVECs)	Inhibition of glucose-induced increases in lactate and ATP, increase in inosine concentrations, reduction in pyruvate concentrations under TNFα treatment, inhibition of adenosine deaminase, xanthine oxidase, and 5′nucleotidase (CD73) activities	[9]
Resveratrol	Purified stilbene	Anti-inflammatory effects, enhancement of glucose metabolism	HepG2 cells	Reduction in expression of NF-kB, IKK-α, IKB-α, and pro-inflammatory cytokines (TNF-α, IL-6, IL-β, COX2); increase in expression of TGFβ1; modulation of glucose metabolism genes (reduction in PEPCK, increase in GCK); regulation of KLF7, HIF1A, and SIRT1 expression	[28]
Procyanidin B2	Apple	Antioxidant, anti-inflammatory effects, protection against ox-LDL-induced injury	In vitro studies on HUVECs, bioinformatics analysis for GSE9647 dataset, THP-1 cell recruitment assay	Alleviation of ox-LDL-induced cell injury, reduction in cell apoptosis, inhibition of LOX-1, MCP-1, and VCAM-1 expression, inhibition of CXCL1/8 expression and THP-1 cell recruitment, reduction in oxidative stress (ROS levels, MDA content, MMP), inhibition of NF-κB activation	[29]
(−)-Epigallocatechin gallate (EGCG) derivatives	Enzymatically prepared from EGCG and vinyl fatty acids	Antioxidant efficacy	Chemical assays (DPPH, ABTS, FRAP, Fe²⁺ chelation), food model (β-carotene bleaching), biological models (LDL and DNA oxidation)	Increased lipophilicity with longer acyl chains influenced antioxidant efficacy through reduction potential, resulting in higher oxidative protection of LDL-c	[30]

**Table 2:** In vivo studies reporting the effects of polyphenols in ameliorating diabetic hallmarks.

Polyphenols	Source	Bioactivities	Model	Mechanisms of Action	Reference
Proanthocyanidin-rich extract	Grape seeds	Anti-diabetic activity Antioxidative properties Renal protective effects	High-fat high-cholesterol diet (HFHCD) + streptozotocin (STZ)-induced type 2 diabetes mellitus (T2DM) in male albino rats	Inhibition of amylase and α-glucosidase activities Improvement of pancreas and Langerhans islets function and structure Alleviation of insulin resistance Reduction of renal inflammatory cytokines (IL-6 and IL-10) Decrease in serum cystatin-C levels Histopathological improvements in kidney, liver, and pancreatic tissues	[10]
Chlorogenic acid, quercetin glycosides, caffeic acid, and procyanidins	Blueberry leaves	Improvement of glucose homeostasis Enhancement of insulin sensitivity Antioxidant activity	High-fat diet (HFD)-induced obesity and diabetes in C57BL/6J mice	Reduction in glucose tolerance, body weight, and plasma glucose levels Decrease in glycated hemoglobin, insulin, triglyceride (TG), and non-esterified fatty acid levels Reduction in pancreatic islet size and insulin content Increase in mRNA levels of pancreatic β-cell proliferation-related genes (Ngn3, MafA, Pax4, Ins1, Ins2) Increase in pancreatic insulin signaling-related genes (IRS-1, IRS-2, PIK3ca, PDK1, PKCε, GLUT-2) Decrease in β-cell apoptosis-related gene (FoxO1) expression Inhibition of triacylglycerol synthesis and enhancement of lipid utilization in liver and white adipose tissue (WAT) Promotion of β-cell proliferation and insulin signaling by chlorogenic acid in pancreatic MIN6 β-cells	[11]
Chlorogenic acid	Tea leaves, roasted green beans, coffee, cocoa, berry fruits, apples, citrus fruits, pears	Antihyperglycemic activity Hepatoprotective effects Antiatherogenic effects	In silico and in vitro studies Streptozotocin (STZ)-induced diabetic rats	Inhibition of carbohydrate metabolizing enzymes (α-amylase and α-glucosidase) Significant reduction in blood glucose, total cholesterol, triglycerides, and other biochemical markers associated with diabetic complications Improvement in body weight, serum HDL-cholesterol, total protein, and albumin levels Betterment in atherogenic indices related to diabetes-associated cardiovascular risks	[61]
Phenolic extract containing luteoforol and *p*-coumaric acid	Mulberry leaves	Hypoglycemic effect Improvement of insulin resistance	In vitro digestion model coupled with Caco-2 monolayer Caco-2/insulin-resistant HepG2 co-culture model	Higher absorption capacity of phenolic acids compared to flavonoids Inhibition of sucrase and maltase activities Decrease in glucose uptake and mRNA expression of glucose transporters (SGLT1, GLUT2, and sucrase-isomaltase) in Caco-2 monolayers Regulation of glucose metabolism by up-regulating mRNA expressions of IRS1, Akt, and GYS2, and down-regulating GSK-3β, PEPCK, and FOXO1 in Caco-2/insulin-resistant HepG2 co-culture model	[62]
Protocatechuic acid	Purified phenolic acid	Improvement of insulin resistance Amelioration of obesity-related glucose and lipid dysregulation	High-fat diet (HFD)-induced obesity and insulin resistance in C57BL/6 mice	Enhanced fatty acid mobilization and utilization Reduction of ectopic lipid accumulation Promotion of hepatic and peripheral insulin action Improvement in systemic insulin resistance as evidenced by hyperinsulinemic-euglycemic mouse clamp	[63]
Gallic acid and *p*-coumaric acids	Isolated compounds	Reduction of cardiovascular risk index 2	Diabetic rats	Reduction of total cholesterol and increase of HDL-c	[64]
Quercetin, gallic, vanillic, and chlorogenic acids	*Opuntia fícus indica* fruit extract	Improvement of antioxidant status and lipid profile	Atherosclerotic Winstar rats fed a high-fat diet	Downregulation of dual oxidases expression, upregulation of Nrf2 pathway	[65]
Epicatechin, procyanidin B1	Cocoa products	Reduction of cardiovascular risk	Human subjects	Increased levels of HDL-c, decreasing levels of glucose and pro-inflammatory cytokines	[66]
Osmudacetone, hispidin, davallialactone, 2,5-bis(4,7-dihydroxy-8-methyl-2-oxo-2H-chromen-3-yl) cyclohexa-2,5-diene-1,4-dione, hypholomin B, and inoscavin A	*Phellinus baumii* extract	Anti-inflammatory effects	ICR male mice and RAW264.7 macrophages	Improvement of insulin sensitivity and glucose metabolism, reduction of total and LDL-c cholesterol and pro-inflammatory cytokines by upregulating the IRS1/PI3K/AKT pathway	[67]
*p*-Hydroxybenzoic acid, ferulic acid, and ethyl ferulate	Vinegar	Reduction of dyslipidemia, anti-inflammatory effects, and gut health promotion	Diabetic mice fed a high-fat diet	Reduction of blood glucose, total cholesterol, and LDL-c. Improvement of HDL-c levels. Inhibition of TLR4/NF-κB pathway, reduction of pro-inflammatory cytokines. Upregulation of probiotic bacteria and downregulation of pathogenic bacteria in the gut	[68]
Oleuropein, salicylic acid, rutin, and *p*-hydroxybenzoic acid	Olive leaf	The combined administration of metformin and olive leaf promoted improved blood glucose levels and reduced dyslipidemia.	Diabetic rats	Better levels of glycated hemoglobin and restoration of normal levels of total cholesterol, LDL-c, and HDL-c	[69]

## Data Availability

Data sharing is not applicable.

## References

[1] Tabák A.G., Herder C., Rathmann W., Brunner E.J., Kivimäki M. (2012). Prediabetes: A high-risk state for diabetes development. Lancet.

[2] Ahmad E., Lim S., Lamptey R., Webb D.R., Davies M.J. (2022). Type 2 diabetes. Lancet.

[3] Romani A., Ieri F., Urciuoli S., Noce A., Marrone G., Nediani C., Bernini R. (2019). Health effects of phenolic compounds found in extra-virgin olive oil, by-products, and leaf of *Olea europaea* L.. Nutrients.

[4] Dias T., Alves M.G., Casal S., Oliveira P.F., Silva B.M. (2017). Promising potential of dietary (poly)phenolic compounds in the prevention and treatment of diabetes mellitus. Curr. Med. Chem..

[5] Kasprzak-Drozd K., Oniszczuk T., Stasiak M., Oniszczuk A. (2021). Beneficial effects of phenolic compounds on gut microbiota and metabolic syndrome. Int. J. Mol. Sci..

[6] Salahuddin M.A.H., Ismail A., Kassim N.K., Hamid M., Ali M.S.M. (2020). Phenolic profiling and evaluation of in vitro antioxidant, α-glucosidase and α-amylase inhibitory activities of Lepisanthes fruticosa (Roxb) Leenh fruit extracts. Food Chem..

[7] Yu M., Zhu S., Huang D., Tao X., Li Y. (2024). Inhibition of starch digestion by phenolic acids with a cinnamic acid backbone: Structural requirements for the inhibition of α-amylase and α-glucosidase. Food Chem..

[8] Saji N., Francis N., Schwarz L.J., Blanchard C.L., Santhakumar A.B. (2020). The antioxidant and anti-inflammatory properties of rice bran phenolic extracts. Foods.

[9] Ozyel B., Le Gall G., Needs P.W., Kroon P.A. (2021). Anti-inflammatory effects of quercetin on high-glucose and pro-inflammatory cytokine challenged vascular endothelial cell metabolism. Mol. Nutr. Food Res..

[10] Ashour M.A., El-Bawab E.M.E., Atty M.E.A.A., Abdallah A.M. (2023). Application of bioactive compounds in grape seed, proanthocyanidins as anti-inflammatory in diabetic rats. J. Glob. Sci. Res..

[11] Li H., Park H.M., Ji H.S., Han J., Kim S.K., Park H.Y., Jeong T.S. (2020). Phenolic-enriched blueberry-leaf extract attenuates glucose homeostasis, pancreatic β-cell function, and insulin sensitivity in high-fat diet–induced diabetic mice. Nutr. Res..

[12] DeFronzo R.A., Ferrannini E., Groop L., Henry R.R., Herman W.H., Holst J.J., Weiss R. (2015). Type 2 diabetes mellitus. Nat. Rev. Dis. Primers.

[13] DiMeglio L.A., Evans-Molina C., Oram R.A. (2018). Type 1 diabetes. Lancet.

[14] Ehrmann D., Kulzer B., Roos T., Haak T., Al-Khatib M., Hermanns N. (2020). Risk factors and prevention strategies for diabetic ketoacidosis in people with established type 1 diabetes. Lancet Diabetes Endocrinol..

[15] Rabiee A., Krüger M., Ardenkjær-Larsen J., Kahn C.R., Emanuelli B. (2018). Distinct signalling properties of insulin receptor substrate (IRS)-1 and IRS-2 in mediating insulin/IGF-1 action. Cell. Signal..

[16] Halim M., Halim A. (2019). The effects of inflammation, aging and oxidative stress on the pathogenesis of diabetes mellitus (type 2 diabetes). Diabetes Metab. Syndr. Clin. Res. Rev..

[17] Lin L.K., Sun Y., Heng B.H., Chew D.E.K., Chong P.N. (2017). Medication adherence and glycemic control among newly diagnosed diabetes patients. BMJ Open Diabetes Res. Care.

[18] Zafar A., Alruwaili N.K., Panda D.S., Imam S.S., Alharbi K.S., Afzal M., Alshehri S. (2021). Potential of natural bioactive compounds in management of diabetes: Review of preclinical and clinical evidence. Curr. Pharmacol. Rep..

[19] Shahidi F., Yeo J. (2018). Bioactivities of phenolics by focusing on suppression of chronic diseases: A Review. Int. J. Mol. Sci..

[20] Shahidi F., Danielski R., Rhein S.O., Meisel L.A., Fuentes J., Speisky H., de Camargo A.C. (2022). Wheat and rice beyond phenolic acids: Genetics, identification database, antioxidant properties, and potential health effects. Plants.

[21] Wojdyło A., Nowicka P., Carbonell-Barrachina Á.A., Hernández F. (2016). Phenolic compounds, antioxidant and antidiabetic activity of different cultivars of *Ficus carica* L. fruits. J. Funct. Foods.

[22] Danielski R., Shahidi F. (2024). Nutraceutical potential of underutilized tropical fruits and their byproducts: Phenolic profile, antioxidant capacity, and biological activity of Jerivá (*Syagrus romanzoffiana*) and Butiá (*Butia catarinensis*). J. Agric. Food Chem..

[23] Shahidi F., Peng H. (2018). Bioaccessibility and bioavailability of phenolic compounds. J. Food Bioact..

[24] Mohamedshah Z., Chadwick-Corbin S., Wightman J.D., Ferruzzi M.G. (2020). Comparative assessment of phenolic bioaccessibility from 100% grape juice and whole grapes. Food Funct..

[25] Li Y., Qian F., Cheng X., Wang D., Wang Y., Pan Y., Tian Y. (2023). Dysbiosis of oral microbiota and metabolite profiles associated with type 2 diabetes mellitus. Microbiol. Spectr..

[26] Abdelli I., Benariba N., Adjdir S., Fekhikher Z., Daoud I., Terki M., Ghalem S. (2021). In silico evaluation of phenolic compounds as inhibitors of α-amylase and α-glucosidase. J. Biomol. Struct. Dyn..

[27] Ezeako E.C., Nworah F.N., Osuji D.O. (2023). Phytocompounds, antioxidant potential, and inhibitory actions of ethanolic leaf fraction of *Sida linifolia Linn. (Malvaceae)* on enzymes linked to inflammation, diabetes, and neurological disorders. Future J. Pharm. Sci..

[28] Tshivhase A.M., Matsha T., Raghubeer S. (2024). Resveratrol attenuates high glucose-induced inflammation and improves glucose metabolism in HepG2 cells. Sci. Rep..

[29] Yuan L., Fan L., Zhang Z., Huang X., Liu Q., Zhang Z. (2023). Procyanidin B2 alleviates oxidized low-density lipoprotein-induced cell injury, inflammation, monocyte chemotaxis, and oxidative stress by inhibiting the nuclear factor kappa-B pathway in macrophages. Phytomedicine.

[30] Peng H., Shahidi F. (2022). Enzymatic synthesis and antioxidant activity of mono-and diacylated epigallocatechin gallate and related by-products. J. Agric. Food Chem..

[31] Li X., Bai Y., Jin Z., Svensson B. (2022). Food-derived non-phenolic α-amylase and α-glucosidase inhibitors for controlling starch digestion rate and guiding diabetes-friendly recipes. LWT.

[32] Ali Asgar M.D. (2013). Anti-diabetic potential of phenolic compounds: A review. Int. J. Food Prop..

[33] Liu H., Zhang M., Ma Q., Tian B., Nie C., Chen Z., Li J. (2020). Health beneficial effects of resistant starch on diabetes and obesity via regulation of gut microbiota: A review. Food Funct..

[34] Ren J., Dai J., Chen Y., Wang Z., Sha R., Mao J. (2024). Physiochemical characterization and ameliorative effect of rice resistant starch modified by heat-stable α-amylase and glucoamylase on the gut microbial community in T2DM mice. Food Funct..

[35] Riyaphan J., Pham D.C., Leong M.K., Weng C.F. (2021). In silico approaches to identify polyphenol compounds as α-glucosidase and α-amylase inhibitors against type-II diabetes. Biomolecules.

[36] Miller N., Joubert E. (2022). Critical assessment of in vitro screening of α-glucosidase inhibitors from plants with acarbose as a reference standard. Planta Med..

[37] Pinaffi A.C.D.C., Sampaio G.R., Soares M.J., Shahidi F., de Camargo A.C., Torres E.A. (2020). Insoluble-bound polyphenols released from guarana powder: Inhibition of alpha-glucosidase and proanthocyanidin profile. Molecules.

[38] Simsek M., Quezada-Calvillo R., Ferruzzi M.G., Nichols B.L., Hamaker B.R. (2015). Dietary phenolic compounds selectively inhibit the individual subunits of maltase-glucoamylase and sucrase-isomaltase with the potential of modulating glucose release. J. Agric. Food Chem..

[39] Aleixandre A., Gil J.V., Sineiro J., Rosell C.M. (2022). Understanding phenolic acids inhibition of α-amylase and α-glucosidase and influence of reaction conditions. Food Chem..

[40] Zhu J., Chen C., Zhang B., Huang Q. (2020). The inhibitory effects of flavonoids on α-amylase and α-glucosidase. Crit. Rev. Food Sci. Nutr..

[41] Sun L., Wang Y., Miao M. (2020). Inhibition of α-amylase by polyphenolic compounds: Substrate digestion, binding interactions and nutritional intervention. Trends Food Sci. Technol..

[42] Du X., Myracle A.D. (2018). Fermentation alters the bioaccessible phenolic compounds and increases the alpha-glucosidase inhibitory effects of aronia juice in a dairy matrix following in vitro digestion. Food Funct..

[43] Alongi M., Celayeta J.M.F., Vriz R., Kinsella G.K., Rulikowska A., Anese M. (2021). In vitro digestion nullified the differences triggered by roasting in phenolic composition and α-glucosidase inhibitory capacity of coffee. Food Chem..

[44] Ziolkowska S., Binienda A., Jabłkowski M., Szemraj J., Czarny P. (2021). The interplay between insulin resistance, inflammation, oxidative stress, base excision repair and metabolic syndrome in nonalcoholic fatty liver disease. Int. J. Mol. Sci..

[45] Chen L., Teng H., Jia Z., Battino M., Miron A., Yu Z., Xiao J. (2018). Intracellular signaling pathways of inflammation modulated by dietary flavonoids: The most recent evidence. Crit. Rev. Food Sci. Nutr..

[46] Ju Z., Li M., Xu J., Howell D.C., Li Z., Chen F.E. (2022). Recent development on COX-2 inhibitors as promising anti-inflammatory agents: The past 10 years. Acta Pharm. Sin. B.

[47] Girard D., Vandiedonck C. (2022). How dysregulation of the immune system promotes diabetes mellitus and cardiovascular risk complications. Front. Cardiovasc. Med..

[48] Tanase D.M., Gosav E.M., Neculae E., Costea C.F., Ciocoiu M., Hurjui L.L., Serban I.L. (2020). Role of gut microbiota on onset and progression of microvascular complications of type 2 diabetes (T2DM). Nutrients.

[49] Afonso A.F., Pereira O.R., Cardoso S.M. (2020). Health-promoting effects of Thymus phenolic-rich extracts: Antioxidant, anti-inflammatory and antitumoral properties. Antioxidants.

[50] Son T.H. (2022). Bioactivity and toxicity evaluation of nutraceuticals using in vitro cell-based models: A review. Vietnam. J. Food Control..

[51] Zhang H., Liu R., Tsao R. (2016). Anthocyanin-rich phenolic extracts of purple root vegetables inhibit pro-inflammatory cytokines induced by H_2_O_2_ and enhance antioxidant enzyme activities in Caco-2 cells. J. Funct. Foods.

[52] Sergent T., Piront N., Meurice J., Toussaint O., Schneider Y.J. (2010). Anti-inflammatory effects of dietary phenolic compounds in an In vitro model of inflamed human intestinal epithelium. Chem. Biol. Interact..

[53] Gu I., Brownmiller C., Stebbins N.B., Mauromoustakos A., Howard L., Lee S.O. (2020). Berry phenolic and volatile extracts inhibit pro-inflammatory cytokine secretion in LPS-stimulated RAW264.7 cells through suppression of NF-κB signaling pathway. Antioxidants.

[54] Martin S.S., Blumenthal R.S., Miller M. (2012). LDL cholesterol: The lower the better. Med. Clin..

[55] Higashi Y. (2023). Endothelial Function in dyslipidemia: Roles of LDL-cholesterol, HDL-cholesterol and triglycerides. Cells.

[56] Xepapadaki E., Nikdima I., Sagiadinou E.C., Zvintzou E., Kypreos K.E. (2021). HDL and type 2 diabetes: The chicken or the egg?. Diabetologia.

[57] Yeo J., Shahidi F. (2020). Suppressing the oxidation of LDL and DNA strand breakage of bioactives in dehulled and hull fraction of lentils. J. Food Bioact..

[58] Albishi T., Banoub J.H., de Camargo A.C., Shahidi F. (2019). Wood extracts as unique sources of soluble and insoluble-bound phenolics: Reducing power, metal chelation and inhibition of oxidation of human LDL-cholesterol and DNA strand scission. J. Food Bioact..

[59] Zeb A. (2020). Concept, mechanism, and applications of phenolic antioxidants in foods. J. Food Biochem..

[60] Wu C.H., Yeh C.T., Yen G.C. (2010). Epigallocatechin gallate (EGCG) binds to low-density lipoproteins (LDL) and protects them from oxidation and glycation under high-glucose conditions mimicking diabetes. Food Chem..

[61] Singh A.K., Rana H.K., Singh V., Yadav T.C., Varadwaj P., Pandey A.K. (2021). Evaluation of antidiabetic activity of dietary phenolic compound chlorogenic acid in streptozotocin induced diabetic rats: Molecular docking, molecular dynamics, in silico toxicity, in vitro and in vivo studies. Comput. Biol. Med..

[62] Zhao Q., Yang J., Li J., Zhang L., Yan X., Yue T., Yuan Y. (2024). Hypoglycemic effect and intestinal transport of phenolics-rich extract from digested mulberry leaves in Caco-2/insulin-resistant HepG2 co-culture model. Food Res. Int..

[63] Xiang Y., Huang R., Wang Y., Han S., Qin X., Li Z., Yang G. (2023). Protocatechuic Acid Ameliorates High Fat Diet-Induced Obesity and Insulin Resistance in Mice. Mol. Nutr. Food Res..

[64] Aslam N., Faisal M.N., Khan J.A., Majeed W. (2022). *Opuntia ficus indica* (L.) fruit extract alleviates oxidative stress through activation of dual oxidases and Keap1/Nrf2 signaling cascades in high-fat-diet associated atherosclerosis rats. Toxicol. Res..

[65] Hybertson B.M., Gao B., McCord J.M. (2022). Effects of the phytochemical combination PB123 on Nrf2 Activation, gene expression, and the cholesterol pathway in HepG2 cells. OBM Integr. Complement. Med..

[66] Yang K., Zhang S., Geng Y., Tian B., Cai M., Guan R., Sun P. (2021). Anti-inflammatory properties In vitro and hypoglycaemic effects of phenolics from cultivated fruit body of *Phellinus baumii* in type 2 diabetic mice. Molecules.

[67] Yang K., Zhang S., Ying Y., Li Y., Cai M., Guan R., Sun P. (2020). Cultivated fruit body of *Phellinus baumii*: A potentially sustainable antidiabetic resource. ACS Omega.

[68] Mansour H.M., Zeitoun A.A., Abd-Rabou H.S., El Enshasy H.A., Dailin D.J., Zeitoun M.A., El-Sohaimy S.A. (2023). Antioxidant and anti-diabetic properties of olive (*Olea europaea*) leaf extracts: In vitro and in vivo evaluation. Antioxidants.

[69] Sarkar A., Chakrabarti A., Bhaumik S., Debnath B., Singh S.S., Ghosh R., Debnath S. (2024). Parkia javanica Edible Pods Reveal Potential as an Anti-Diabetic Agent: UHPLC-QTOF-MS/MS-Based Chemical Profiling, In Silico, In Vitro, In Vivo, and Oxidative Stress Studies. Pharmaceuticals.

[70] Zatterale F., Longo M., Naderi J., Raciti G.A., Desiderio A., Miele C., Beguinot F. (2020). Chronic adipose tissue inflammation linking obesity to insulin resistance and type 2 diabetes. Front. Physiol..

[71] Ellulu M.S., Patimah I., Khaza’ai H., Rahmat A., Abed Y. (2017). Obesity and inflammation: The linking mechanism and the complications. Arch. Med. Sci..

[72] Youl E., Bardy G., Magous R., Cros G., Sejalon F., Virsolvy A., Oiry C. (2010). Quercetin potentiates insulin secretion and protects INS-1 pancreatic β-cells against oxidative damage via the ERK1/2 pathway. Br. J. Pharmacol..

[73] Mahendra V.P., Haware D.J., Kumar R. (2019). cAMP-PKA dependent ERK1/2 activation is necessary for vanillic acid potentiated glucose-stimulated insulin secretion in pancreatic β-cells. J. Funct. Foods.

[74] Manzano M., Giron M.D., Vilchez J.D., Sevillano N., El-Azem N., Rueda R., Lopez-Pedrosa J.M. (2016). Apple polyphenol extract improves insulin sensitivity in vitro and in vivo in animal models of insulin resistance. Nutr. Metab..

[75] Kawakami Y., Watanabe Y., Mazuka M., Yagi N., Sawazaki A., Koganei M., Natsume M., Kuriki K., Morimoto T., Asai T. (2021). Effect of cacao polyphenol-rich chocolate on postprandial glycemia, insulin, and incretin secretion in healthy participants. Nutrition.

[76] Liu S., Yu J., Fu M., Wang X., Chang X. (2021). Regulatory effects of hawthorn polyphenols on hyperglycemic, inflammatory, insulin resistance responses, and alleviation of aortic injury in type 2 diabetic rats. Food Res. Int..

[77] Yu C., Xiao J.H. (2021). The Keap1-Nrf2 system: A mediator between oxidative stress and aging. Oxid. Med. Cell. Longev..

[78] Rakshe P.S., Dutta B.J., Chib S., Maurya N., Singh S. (2024). Unveiling the Interplay of AMPK/SIRT1/PGC-1α axis in Brain Health: Promising Targets Against Aging and NDDs. Ageing Res. Rev..

[79] Abdel-Moneim A., Abd El-Twab S.M., Yousef A.I., Reheim E.S.A., Ashour M.B. (2018). Modulation of hyperglycemia and dyslipidemia in experimental type 2 diabetes by gallic acid and *p*-coumaric acid: The role of adipocytokines and PPARγ. Biomed. Pharmacother..

[80] Sarriá B., Gomez-Juaristi M., López S.M., Cordero J.G., Bravo L., Briz M.R.M. (2020). Cocoa colonic phenolic metabolites are related to HDL-cholesterol raising effects and methylxanthine metabolites and insoluble dietary fibre to anti-inflammatory and hypoglycemic effects in humans. PeerJ.

[81] Xia T., Zhang Z., Zhao Y., Kang C., Zhang X., Tian Y., Wang M. (2022). The anti-diabetic activity of polyphenols-rich vinegar extract in mice via regulating gut microbiota and liver inflammation. Food Chem..

